# Identifying the Factors Contributing to the Severity of Truck-Involved Crashes in Shanghai River-Crossing Tunnel

**DOI:** 10.3390/ijerph17093155

**Published:** 2020-05-01

**Authors:** Shengdi Chen, Shiwen Zhang, Yingying Xing, Jian Lu

**Affiliations:** 1 School of Transport & Communications, Shanghai Maritime University, Shanghai 201306, China; sdchen@shmtu.edu.cn; 2 Shanghai Key Laboratory of Rail Infrastructure Durability and System Safety, Key Laboratory of Road and Traffic Engineering of the State Ministry of Education, College of Transportation Engineering, Tongji University, Shanghai 201804, China; yingying199004@tongji.edu.cn (Y.X.); jianjohnlu@tongji.edu.cn (J.L.)

**Keywords:** truck crashes injury propensity, tunnel traffic crashes, risk factors, ordered logit model

## Abstract

The impact that trucks have on crash severity has long been a concern in crash analysis literature. Furthermore, if a truck crash happens in a tunnel, this would result in more serious casualties due to closure and the complexity of the tunnel. However, no studies have been reported to analyze traffic crashes that happened in tunnels and develop crash databases and statistical models to explore the influence of contributing factors on tunnel truck crashes. This paper summarizes a study that aims to examine the impact of risk factors such as driver factor, environmental factor, vehicle factor, and tunnel factor on truck crashes injury propensity based on tunnel crashes data obtained from Shanghai, China. An ordered logit model was developed to analyze injury crashes and property damage only crashes. The driver factor, environmental factor, vehicle factor, and tunnel factor were explored to identify the relationship between these factors and crashes and the severity of crashes. Results show that increased injury severity is associated with driver factors, such as male drivers, older drivers, fatigue driving, drunkenness, safety belt used improperly, and unfamiliarity with vehicles. Late night (00:00–06:59) and afternoon rushing hours (16:30–18:59), weekdays, snow or icy road conditions, combination truck, overload, and single vehicle were also found to significantly increase the probability of injury severity. In addition, tunnel factors including two lanes, high speed limits (≥80 km/h), zone 3, extra-long tunnels (over 3000 m) are also significantly associated with a higher risk of severe injury. So, the gender, age of driver, mid-night to dawn and afternoon peak hours, weekdays, snowy or icy road conditions, the interior zone of a tunnel, the combination truck, overloaded trucks, and extra-long tunnels are associated with higher crash severity. Identification of these contributing factors for tunnel truck crashes can provide valuable information to help with new and improved tunnel safety control measures.

## 1. Introduction

Trucks are critical to freight logistics and have an effect on the economic well-being of a nation. Meanwhile, there are a lot of unique characteristics of trucks that would affect crash severity, such as high gross vehicle weight, long vehicle length, and poor stopping distance, which could lead to enormous casualties and property losses. According to the statistics of Traffic Management Bureau of The Public Security Ministry, 25,000 people were killed and another 46,800 were injured in China in 50,400 crashes involving trucks in 2016, accounting for 48.23%, 27.81%, and 30.5% of the total traffic crashes, respectively. The ratio of fatal truck crashes (48.23%) in total traffic crashes is much higher than the ratio of trucks (12.28%) in total vehicles. This shows that there is a serious safety problem involving trucks, based on these statistics. Furthermore, the closed environment of road tunnels not only hinders smoke and heat dissipation, but also hampers rescue operations by firefighters and makes it difficult to evacuate [1,2]. Therefore, if a crash happens involving a truck in a tunnel, the consequence would be more serious and cause more casualties. This is indicated by three previous disastrous tunnel traffic crashes (i.e., Mont Blanc tunnel from France to Italy in 1999, the St. Gotthart tunnel of Switzerland in 2001, and Yanhou Tunnel of China in 2014), which led to the death of more than 80 people in total [2,3,4]. Thus, truck crashes have not only raised awareness among the public as well as the government on the safety aspect of trucks in tunnels, but have also attracted a number of scholars to explore truck safety problems.

## 2. Literature Review

The analysis of trucks is an important issue in crashes. The existing researches on truck-involved crashes mainly focus on these aspects: crash frequencies and injury severity as well as their respective risk factor analyses [5]. In recent years, not only crash frequencies (or rates), but injury severity of truck drivers has also been a research hotspot. Regression analysis, particularly logit-based and ordered probability regression models, were commonly used in the literature to identify the factors affecting injury severity in truck crashes [6,7,8,9,10,11,12,13,14]. For instance, Duncan et al. (1998) analyzed the effect of the risk factors on injury severity of passenger car occupants involved in truck-passenger-car rear-end collisions using ordered probit models [6]. Khorashadi et al. (2005) explored the difference between rural and urban driver-injury severities in large truck-involved crashes using multinomial logit models. The results showed that 13 variables were found to be significant in rural but not urban areas, and 17 variables significantly influenced injury severity in urban but not rural areas [7]. Lemp et al. (2011) explored causal factors affecting injury severities of large truck crashes using heteroskedastic ordered probit models. Results suggested that the number of trailers increased the likelihood of fatalities and severe injury, but the truck length and gross vehicle weight rating decreased the injury risk [10]. Chang and Chien (2013) also explored the factors affecting injury severity of truck crashes by using a non-parametric classification tree model. The results showed that drunk driving, safety belt use, contributing circumstance and driver/vehicle action, vehicle type, collision type, number of vehicles, and crash location were the key factors determining injury severity in truck-involved crashes [12]. Dong et al. (2017) analyzed the influences of risk factors on the frequency and severity of large truck-involved crashes using multinomial logit (MNL) and negative binomial (NB) models. They found that the severity and frequency of crashes increases significantly when large trucks are involved [14].

As the literature showed above, most of the existing studies focused on the severity of truck-involved crashes on highways and little research analyzed the impact of the risk factors on injury severity in river-crossing tunnel crashes involving trucks. However, tunnel traffic crashes involving trucks are also one of the emphases for tunnel researches. Many studies’ efforts in this field have been allocated to crash frequencies analysis. Based on data from a police crash database, Lemke (2000) analyzed average crash rates and crash cost rates for different cross sections of tunnels on German roads. Results indicate that crash rates are lower for roads in tunnels [15]. Nussbaumer (2007) investigated tunnel crashes that occurred in 50 Austrian tunnels based on a police crash database. It was found that crash severity in tunnels was greater than on open roadways [16]. Amundsen and Engebretsen (2009) analyzed traffic crashes that occurred in Norwegian road tunnels from 2001 to 2006. They divided the tunnels into four zones to refer to (Table 1) and found that the crash rates were higher in the entrance zone (zone 1 and zone 2) than other zones and the severity of crashes were higher in tunnels than on stretches of road [17]. Using data derived from police records, Ma et al. (2009) carried out crash analysis for four freeway tunnels of China from 2003 to 2004 [18]. Based on incident records provided by the Land Transport Authority, Yeung and Wong (2013) investigated tunnel traffic crashes that occurred in three Singapore expressway tunnels over 2009–2011 [19]. They divided each road tunnel into three zones and analyzed crash characteristics for each zone. The results showed that crash rates in the interior zones were lower than in transition zones (zone 1 and zone 2, refer to Table 1), while traffic crashes in interior zones were more likely to result in casualties than in transition zones. Jiang et al. (2016) identified 13 factors that contributed to escaping after crashes in Shanghai river-crossing tunnels and they found that a perpetrator’s tendency to leave the crash scene without reporting a crash was higher at night, in the tunnel exit, near to or in short tunnels, when a two-wheeled vehicle or heavy goods vehicle was involved, and when alcohol was involved [20]. Caliendo et al. (2016) analyzed severe crashes (fatal and injury crashes only) that occurred in 260 Italian road tunnels by using a random-parameters regression model [21]. Lu et al. (2017) analyzed the risk factors affecting the injury severity of traffic crashes at Shanghai river-crossing tunnel by using data extracted from Shanghai Transport and Port Authority crash incident files [22]. Results indicated that several contributing factors were associated with higher severity crashes, such as four or more lanes, extra-long tunnels (over 3000 m), interior zones, and the maximum longitudinal gradient of tunnels. Above all, in this study, the four tunnels were segmented into four zones (Table 1) and both the highest crash and casualty rates happened in Zone 3.

Thus, based on previous literatures, with the type of truck as the main research object involved in crashes, four factors will be explored to identify the relationships among them and also the measures of improving truck safety and avoiding crashes are provided in this research. Therefore, in order to reduce the truck-involved crashes in tunnel zones, the ordered logit model and statistical method were used to identify these factors such as driver factor, environmental factor, vehicle factor, and tunnel factor. This study aims to reduce the severity of tunnel traffic crashes, especially truck crashes, which could be severe crashes. Due to the statistical data based on police incident records, it should formulate relevant measures to avoid traffic crashes and reduce the severity of crash consequences to a greater extent. It provides a basis for reducing large vehicle traffic crashes in tunnels.

Overall, the literature on the severity of tunnel traffic crashes involving trucks appears to be limited. To fill this gap, a study is conducted to identify the factors contributing to the severity of truck involved crashes in tunnels. Specifically, a representative sample of truck crashes in six river-crossing tunnels in Shanghai over 2014–2016 is examined to determine the factors affecting the injury severity of these crashes. Several potential risk factors including driver, environment vehicle, site, and tunnel factors are considered in the study.

## 3. Materials and Methods

### 3.1. Database

By convention, crash data extracted from police incident records should be relatively reliable. Previous studies on tunnel traffic crashes in China were carried out based on police incident records [18,23]. In this study, crashes involving trucks were derived from the police database provided by Shanghai Transport and Port Authority. Based on the variable of “vehicle type”, only crash records involving at least one truck were selected for this study. After a selection of crash records, there were 1813 truck-involved crashes in total occurring in the river-crossing tunnel in Shanghai during the three-year period. Each crash sample contains characteristics of drivers, crash location, crash time, vehicle features, environmental conditions, and degree of casualties. Therefore, the factors affecting the injury severity are broadly classified into (1) driver factors (age, gender, alcohol, safety belt, and driver fatigue), (2) environmental factors (including weather and time), (3) vehicle factors (overload or not, single or above), and (4) tunnels factors (tunnel length).

### 3.2. Risk Factors

#### 3.2.1. Driver Factor

Five driver factors were included in the data. Driver age was classified into three groups based on driving habit and crash involvements: ≤25, 26–64, and ≥65. Drivers’ gender, alcohol, and safety belt use were binary variables and were considered as potential risk factors in the study. In this paper, safety belt used improperly means one of the following conditions: (1) safety belt used improperly; (2) shoulder belt only; (3) lap belt only. Furthermore, not wearing a seat belt also belongs to safety belt used improperly. According to the Implementation Regulations of National Highway Traffic Safety Law of the People’s Republic of China [24], drivers should rest for at least 20 min after driving continuously for 4 h, otherwise it is fatigue driving. Therefore, based on continuous driving time, driver fatigue could be divided into three groups: fatigue driving, non-fatiguing driving, and unknown. The familiarity of the truck driver with the vehicle is classified into three categories: driven this vehicle ≤10 times in the past 6 months, driven this vehicle >10 times in the past 6 months, and unknown.

#### 3.2.2. Environmental Factor

Whether the crash occurred during the weekend was considered as one of environmental factors. According to previous studies, traffic crashes that occur after 17:00 on Friday are similar to those occurring on Saturday and Sunday [25,26]. Thus, weekends are defined as from 17:00 Friday to 23:59 Sunday. Based on working time patterns and peoples’ lifestyles in Shanghai, time of day was divided into five groups: 00:00–06:59 (midnight to dawn), 07:00–09:29 (morning peak hours), 09:30–16:29 (working hours), 16:30–18:59 (afternoon peak hours), and 19:00–23:59 (afternoon to midnight). Three levels of road surface conditions were assigned: rain, snow and ice, and dry.

#### 3.2.3. Vehicle Factor

Two truck body types were included in the data, and they are single-unit truck and combination truck. In recent years, it was quite common for trucks to overload in our country, thus truck overload was an important factor influencing traffic safety and was classified into two groups: overload and non-overload. Overload refers to the actual load of transportation means exceeding the approved maximum allowable limit. Truck overload usually refers to the motor vehicle transported goods exceeding the loading capacity of truck motor vehicles. The number of vehicles was divided into three groups: single vehicle, two vehicles, and three or more vehicles.

#### 3.2.4. Tunnel Factor

There were several tunnel factors: speed limit, tunnel length, number of lanes, tunnel zones, least horizontal radius, and maximum longitudinal gradient. Since none of the six river-crossing tunnels are less than 2000 m in length, tunnel length could be divided into two types in the light of Code for Design of Road Tunnel (2004) [26], namely long tunnel (3000 ≥ L > 1000 m) and extra-long tunnel (L > 3000 m). The speed limit of the tunnel was categorized into three groups in accordance with the usual speed limit classification in urban and sub-urban areas: <50, 50–79, and ≥80 km/h [27]. The six river-crossing tunnels that were analyzed in this study are all divided into two tubes that carry one-way traffic. Meanwhile, the two tubes of each tunnel have the same number of lanes. As a result, the number of lanes in each tube falls into three categories: two lanes, three lanes, and four lanes or more.

**Figure 1:**
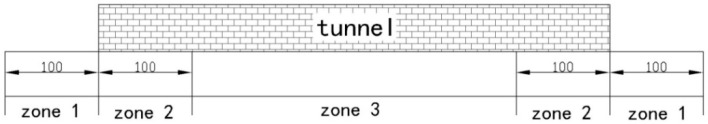
Tunnel Zones.

**Figure 2:**
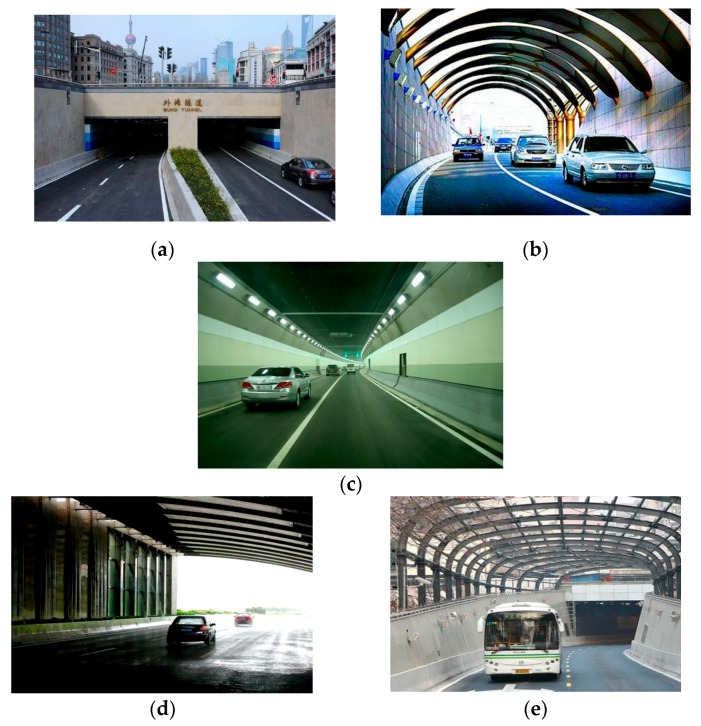
Different Tunnel Zones: (**a**,**e**) Zone 1; (**b**,**d**) Zone 2; (**c**) Zone 3. (Data source: Baidu Picture).

Tunnel zones in this study (Figure 1) are classified into three groups: Zone 1, Zone 2, and Zone 3. Zone 1 and Zone 2 are the transition zones of the tunnel and are defined as the first 100 m in front of the tunnel and the first 100 m into the tunnel, respectively. Zone 3 is the remainder of the tunnel, also known as the interior zone. Tunnels shorter than 200 m have only zones 1 and 2. Figure 2 shows sketch maps of different tunnel zones. Tunnel least horizontal radius and maximum longitudinal gradient are both continuous variables.

In conclusion, the description and levels of these variables are summarized in Table 2. The table also shows the proportional distribution of variables in the different categories.

### 3.3. Statistical Analysis

Injury severity is generally recorded on an ordinal scale [11]. Most commonly, the injury severity of a traffic crash is classified into four ordinal levels: no injury/property damage only (PDO), possible injury (PI), non-incapacitating (NI), and fatal/incapacitating (FI). An ordered logit model is suitable to analyze such data. As a matter of fact, it has been a popular approach for modeling injury severity [28,29,30].

The ordered logit model is based on the cumulative probability of the response variable. Let $y_{i}$ be an ordinal response variable with *M* categories for the *i*th subject, alongside a vector of covariates $x_{i}$, then the cumulative probability for an ordered logit model is expressed in the form [31]:

(1) $$Pr\left(y_{i}\leq j\right)=\sum_{k=1}^{j}Pr\left(y_{i}=k\right)=\frac{1}{1+\exp\left(x_{i\beta }-\alpha_{j}\right)},\;j=1,2,\ldots ,M-1.$$

Given that the severity is an ordered-response discrete variable, an ordered logit model can be written in terms of the cumulative probability of crash injury as:

(2) $$\log\left[\frac{\mathrm{Pr}\left(y_{i}\leq j\right)}{\mathrm{Pr}\left(y_{i}>j\right)}\right]=\alpha_{j}-\beta x_{i},\;j=1,2,\ldots ,M-1$$

where $y_{i}$ is an ordinal injury severity, $x_{i}$ is a vector of independent variables, *j* is a severity category, M is the number of categories of $y_{i}$, *β* is a vector of parameters to be estimated, and $\alpha_{j}$ are cut points for the thresholds of the ordered model. When an independent variable $x_{i}$ increases by one unit as all other factors remain constant, the odds increases by a factor exp(β), which is called the odds ratio (OR) [25]. The ratio is used for estimating the likelihood of the injury severity for different independent variables.

The ordered logit model only applies to data that meet the proportional odds assumption in which the slope coefficients in the model are the same across response categories. Its null hypothesis states that the location parameters (slope coefficients) are the same across response categories. The Brant test is applied to verify whether the proportional odds assumption is valid. The significance level of the Chi-Square statistic provides evidence whether the assumption has been violated. If the null hypothesis is rejected (sig < 0.05), one would conclude that the proportional odds assumption has been violated. If one fails to reject the null hypothesis (sig > 0.05), one concludes that the ordered logit coefficients are equal across the levels of the outcome.

Before modeling crash data, in general, it is necessary to carry out a multicollinearity test. In this study, a multicollinearity test was computed using a backward regression model in which all factors were initially included, and insignificant factors were subsequently removed by the independent variables’ contribution to the dependent variable. Entry and removal probabilities for the stepwise procedure were set at 0.05 and 0.1, respectively. The Variance Inflation Factor (VIF) and tolerance are both widely used for testing multicollinearity [32]. VIF quantifies the severity of multicollinearity and provides an index that measures how much the variance of an estimated regression coefficient is increased because of multicollinearity. A common rule of thumb is that a VIF value in excess of 10 indicates high multicollinearity [32]. Tolerance is the inverse of VIF.

## 4. Results

Before detailed results are presented, preliminary findings point to an important conclusion. The results of multicollinearity tests are shown in Table 3. The VIF of all variables in Table 3 are less than 10, while the tolerance of all variables is greater than 0.1. According to the common rule of thumb, the tests suggest that no explanatory variables are highly correlated.

The ordered logit model was estimated with various combinations of the explanatory variables described in the data selection. Table 4 shows the model tests of parallel lines and Table 5 presents the model estimates. From Table 4, it can be seen that the null hypothesis is accepted (sig > 0.05). Therefore, the ordered logit model is suitable to describe the data in this study. From Table 5, several factors are found to be significant. Drivers’ gender is an important factor associated with crash severity and female drivers (OR = 0.336, 95% CI = 0.157–0.717) are associated with decreased severity of crash. Compared to drivers over 65 years old, young and middle-aged drivers are associated with a lower risk of severe injuries. The tunnel traffic crashes induced by fatigue driving (OR = 1.250, 95% CI = 1.004–1.554) will lead to a higher risk of injury severity than crashes caused by non-fatiguing driving. Alcohol use has a large effect on driving behaviors and traffic safety. The result is certainly intuitive as drivers under the influence of alcohol are more likely to be involved in fatal/incapacitating injuries (OR = 2.401, 95% CI = 1.399–4.116). Besides, compared to using a safety belt improperly, using a safety belt properly can decrease the probability of severe injury (OR = 0.656, 95%CI = 0.433–0.996). The familiarity of the truck driver with the vehicle also has an impact on the severity of crashes involving trucks. To be specific, drivers who have driven a truck more times (OR = 0.795, 95%CI = 0.635–0.995) are less likely to be involved in severe crashes.

The time period from mid-night to dawn (00:00–06:59) (OR = 2.261, 95% CI = 1.443–3.543) exhibits the highest risk for a fatal/incapacitating crash, followed by afternoon rush hours (16:30~18:59) (OR = 1.556, 95% CI = 1.021, 2.370), while the morning peak time and working hours are not found to be significantly associated with crash injury severity. Compared to weekends, weekdays are more likely to be involved in fatal/incapacitating crashes (OR = 1.381, 95% CI = 1.059–1.802). Furthermore, setting dry road surface as the base category, there is an increase in injury propensity on wet road surfaces (OR = 1.452, 95% CI = 1.077–1.960) and snow and icy road conditions (OR = 4.495, 95% CI = 1.297–15.580).

Truck type is identified to be statistically associated with injury severity of the crashes. Crashes involving combination trucks lead to more severe injuries compared to single-unit trucks (OR = 0.812, 95% CI = 0.665–0.992). Truck overloading (OR = 1.242, 95% CI = 1.009–1.528) is associated with higher risk of severe injuries than trucks with no overloading. Compared with three or more vehicles involved in a crash, the involvement of a single vehicle (OR = 2.956, 95% CI = 1.511–5.789) and two vehicles (OR = 1.647, 95% CI = 1.145–2.368) are associated with a higher severity of tunnel traffic crashes.

Compared to a high speed limit (over 79 km per hour), middle speed limits (OR = 0.475, 95% CI = 0.318–0.710) and low speed limits (under 50 km/h) (OR = 0.387, 95% CI = 0.203–0.739) are correlated with decreased injury severity level. Setting four or more lanes as the base category, three lanes (OR = 1.694, 95% CI = 1.160–2.472) and two lanes (OR = 2.024, 95% CI = 1.283–3.190) increase the probability of higher crash severity. Regarding crash location, crashes occurring in zone 1 (OR = 0.728, 95% CI = 0.572–0.925) and zone 2 (OR = 0.421, 95% CI = 0.269–0.656) are less likely to result in severe injuries compared to zone 3. In addition, tunnel length is found to affect the injury severity of the tunnel crashes. The extra-long tunnel (L > 3000 m) contributes to a higher injury risk propensity than long tunnels (1000 < L ≤ 3000 m) (OR = 0.428, 95% CI = 0.309–0.593). Maximum longitudinal gradient and least horizontal radius were not found to be significantly related with tunnel crash severity.

## 5. Discussion

### 5.1. Driver Factor

Model results show that the driver’s gender is significantly associated with injury severity in truck crashes that occurred in river-crossing tunnels. Comparatively, male truck drivers are more likely to be involved in fatal/incapacitating injuries than female truck drivers. This could be attributed to the higher risk-taking behavior of male drivers such as fatigue driving, speeding, drunk driving, and aggressive driving. The result is in line with past studies that male drivers tend to increase the risk of high injury severity [23,26,33,34,35,36,37].

Drivers’ age is another factor that has a significant association with injury severity [38,39,40,41,42,43,44]. Compared to the older truck drivers, younger truck drivers are 53.2% less likely to be involved in severe truck crashes (OR = 0.468) and middle-aged truck drivers are 59.5% less likely to be associated with severe injuries (OR = 0.405). This could be attributed to drivers’ higher injury risk at an older age and a decline in physiological strength and reaction capacity. 

Fatigue in truck drivers is not uncommon [5,45]. Truck drivers often become fatigued because they work long hours, face strenuous deadlines, and have to abide by rigorous schedules. The models developed in this study show that driver fatigue increases the probability of injury severities in river-crossing tunnel crashes. 

In terms of the influence of alcohol, drunk driving is found to be a significant factor related with higher fatality risk, which is consistent with the previous literature [46]. Compared to a normal condition, drunk drivers are more likely to lead to higher injury risk propensity, proving that driving under the influence of alcohol is dangerous for the drivers. This is probably due to their risk-taking behavior and decrease of proper judgement caused by alcohol, which often increases their involvement in more severe injury crashes. Furthermore, drivers under the influence of alcohol also have slower reaction times and poorer vision, which may lead to less deceleration in cases of an emergency.

Generally speaking, safety belts provide effective protection for drivers and reduce injury severity. However, if safety belts are used improperly, they may increase the probability of severe injury. One possible reason for this finding is that drivers would be thrown into the components in the passenger compartment such as the dashboard, wheel, steering column, console, and windshield if they are not wearing their safety belt properly, which may cause greater damage to drivers during the crash phase. In addition, drivers who are not wearing a seatbelt may be thrown out of their vehicle, which results in a higher injury severity level. The finding of this factor is also substantiated by previous studies [47,48,49].

The familiarity of the truck driver with the vehicle was found to have a statistically significant effect. According to the ordered logit model, drivers who have driven a truck fewer times are likely to be involved in more severe crashes. This result is intuitively reasonable and consistent with the literature [50].

### 5.2. Environmental Factors

**Figure 3:**
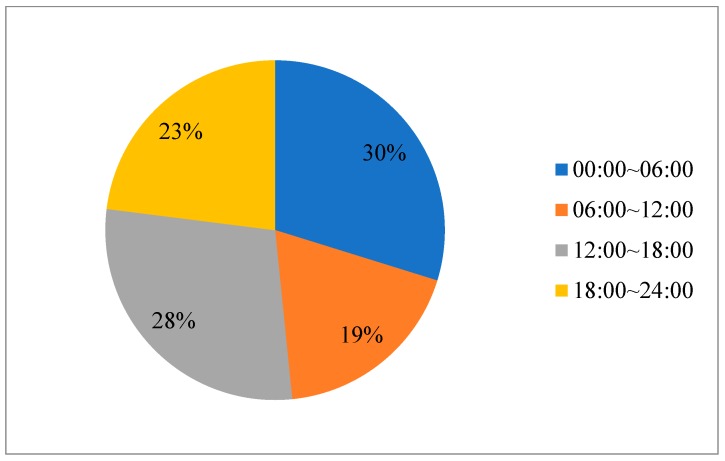
Distribution of fatigue driving by time.

The time of the crash is one of the significant environmental factors identified in the present study. Comparing time from afternoon to midnight (19:00–23:59), crashes that occurred late at night (00:00–06:59) and during afternoon rush hours (16:30–18:59) are statistically associated with a higher injury severity level, especially from midnight to dawn. This is probably due to fatigue driving during these two periods. Truck drivers work in high-intensity conditions, therefore fatigue driving is also very common and a serious problem. The big data of logistics report in China shows that fatigue driving occurring in the afternoon is as serious as that in the early morning, together accounting for nearly 60% of the total (Figure 3). Meanwhile, the most serious period of truck driver’s fatigue driving is between 4 pm and 5 pm, which is exactly the time that truck drivers keep continuously driving for 4 h after starting work at noon. Moreover, lower traffic volume during the night could be related with higher speeds, which are more likely to result in severe injuries [50].

It has been observed that day of the week is significantly associated with injury severity. If a crash occurs on the weekend, the injury severity is less severe. One possible explanation is that there are a higher number of vehicles in the weekdays, which increases the exposure of passenger vehicles in the tunnel. This exposure signifies greater interaction between trucks and passenger vehicles, which may increase the probability of severe crashes. A similar result was found by Islam and Hernandez (2013) [51].

Our results show that there is an obvious relationship between tunnel truck crash severity and road surface conditions. Compared to a dry road surface, snowy or icy road conditions and the presence of rain significantly increase the probability of a fatal/incapacitating injury. The reason for this is because snowy or icy road conditions and the presence of rain build up a thin film of water between the road surface and the tires, which reduces the friction of the road surface in contact with a vehicle’s tires [52,53]. This reduction of friction between the road surface and the tires increases the injury risk, especially at corners [53].

### 5.3. Vehicle Factors

The impact of different types of truck bodies on injury severity was investigated in our study and the results show that the combination truck increases the likelihood of severe injuries. The most likely reason for this is that combination trucks usually have lower acceleration rates and longer vehicle lengths than single-unit trucks. Besides, combination truck drivers may find it difficult to identify other vehicle drivers, particularly smaller vehicles. This could be attributed to the limitations on the truck driver’s view. In China, drivers sit on the left side of the vehicle. Therefore, on the right side of the truck, the driver’s view relies almost exclusively on mirrors. The combination truck width restricts the angular size of the field in the right-hand mirror, while its height and door limit the driver’s direct view of the lane next to the truck [49].

In China, truck overloading is common. Overloaded trucks may cause serious damage to traffic security and pavement structure. The results in this study agree that overloaded trucks bring higher injury severity. This is probably due to the increase of braking distance caused by overload. The more serious the truck overloads, the longer the braking distance is.

The number of vehicles involved in a tunnel truck crash is also an important factor that affects injury severity. When the number of vehicles involved in a crash increases, the level of injury severity decreases. A single-truck crash results in a higher injury propensity than multiple vehicle crashes involving a truck. One possible explanation for this is that multiple vehicle crashes, such as pile-ups, alleviate injury severity resulting from some unforeseen dynamics and preventive technologies present in vehicles [54].

### 5.4. Tunnel Factors

Tunnel factors found to impact the injury severity are presented in Table 5. It is interesting to find that the greater the number of lanes, the less the risk of severe injury crashes. This could be due to better separation of the trucks from other types of vehicles in a multi-lane tunnel (four or more lanes (83.8%) account for most cases in the sample) [11].

In the data sample, over half (52.7%) of the crashes happened at transition zones of the tunnel. However, the results indicate that crashes that happened at an interior zone (zone 3) contribute to a higher injury propensity than transition zones (zone 1 and zone 2). This result is in accordance with previous studies mentioned above [18,19].

The effect of speed limit on injury severity was also conducted in our study. Results show that the probabilities of fatal/incapacitating injury increase with increasing speed limit. These results agree with previous studies [11,49]. Higher speed limits on the road imply higher speeds of the vehicles on the road [37]. Meanwhile, Haleem and Abdel-Aty (2010) found that there was a strong connection between higher vehicle speeds and more severe injury levels [55]. As a consequence, a reduction in the speed limit could help decrease the injury severity by providing drivers with a better chance for maneuvering and braking actions to avoid collisions or lessen their severity. Besides, higher speed limits may lead to an increase of speed variance between different types of vehicles in the tunnel, especially trucks and passenger cars, while the speed variance is generally viewed as having a negative effect on traffic safety.

The modeling results also show that tunnel length is positively associated with increased severity of crash and a longer tunnel results in higher severity injuries, which is consistent with previous findings that crash severity is higher in long tunnels [15,22]. This may be ascribed to the drivers’ diminishing concentration with increasing tunnel length. The impact of horizontal (least horizontal radius) and vertical (maximum longitudinal gradient) alignment of the tunnel were also examined. These were found to be statistically insignificant.

## 6. Conclusions

This study investigates the impact of potential risk factors on injury severity for 1883 truck crashes occurring from 2014 to 2016 in river-crossing tunnels of Shanghai. The ordered logit model was adopted to analyze the injury severity in truck crashes. The risk factors including drivers’ information of male drivers and older age (≥65); environmental conditions of mid-night to dawn (00:00–06:59) and afternoon peak hours (16:30–18:59), weekdays, snow, ice, and rain; vehicle factors of combination truck, single truck, and overloaded truck; and tunnel characteristics of the interior zone (zone 3) high speed limit, 2-lane and 3-lane tunnels, and extra-long tunnels will lead to a higher probability of severity of crash.

In order to avoid truck crashes and decrease the injury severity of crashes, based on the conclusion, drivers’ education about their vehicle and safety driving practices should be reinforced to improve them. The policy could be made according to the research conclusion, as for too many truck crashes in tunnels, the physical and psychological qualities of the drivers of large trucks could be fully considered from the design of the entrance of the tunnel. Tunnel lighting needs to be carefully designed to reduce the effect of “black hole” in tunnel entrances and “white hole” in tunnel exits. Further, poor lighting or over-illumination should be avoided in the interior zone. Furthermore, the construction of protective facilities, such as parking belts, fire exits, emergency evacuation passages, etc. should be focused on. For advice, overloading monitoring and management platforms should be installed to automatically monitor overloaded trucks in real-time, and the marking and lighting equipment also should be improved. At the same time, the relevant data should be provided to the departments for further research. It is necessary to improve the equipment and measures in the tunnel to prevent rear-end crashes of vehicles.

Although many risk factors were analyzed in this study, there are also many other factors (such as traffic volume, truck age, truck driving experience, and driver break time) that may also be related to the injury severity level of truck crashes. For instance, the impact of the seriousness of truck crashes in the case of mixed traffic when the volume is large, the seriousness of crashes when truck-based traffic volume is heavy, and the seriousness of crashes when small-car traffic volume is heavy should also be explored. The impact of the service life of trucks on the severity of traffic crashes, the impact of driver’s driving experience on the severity of crashes, the types of freight or goods that the truck carried on the severity of crashes, etc. should also be studied. Furthermore, a comparative analysis of alternative model structures, subject to the availability of adequate data, should also be identified as an area of future research.

## Figures and Tables

**Table 1:** Definition of Tunnel Zones by Three Studies.

Author	Amundsen and Engebretsen (2000)	Ma et al., (2009)	Yeung et al., (2013)
Zone 1	A distance of 50 m ahead (of the entrance)	100 m ahead	250 m ahead
Zone 2	First 50 m inside (the tunnel)	First 100 m inside	First 250 m inside
Zone 3	Next 100 m (from Zone 2)	Next 300 m	The remainder
Zone 4	The remainder	The remainder	–

**Table 2:** Descriptive Statistics of Variables.

Variables	Codes	Share
Driver’s gender	0—male	98.0%
	1—female	2.0%
Driver’s age	1—≤25	6.4%
	2—26–64	90.3%
	3—≥65	3.3%
Fatigue	1—Unknown	6.0%
	2—Yes	35.1%
	3—No	58.9%
Alcohol	0—Yes	3.9%
	1—No	96.1%
Safety belt used properly	0—Yes	93.7%
	1—No	6.3%
Familiarity with vehicle	1—Unknown	2.2%
	2—Driven this vehicle >10 times in the past three 6 months	70.0%
	3—Driven this vehicle ≤10 times in the past three 6 months	27.9%
Truck body type	0—Single-unit truck	52.2%
	1—Combination truck	47.8%
Overload	0—Yes	35.2%
	1—No	64.8%
Number of vehicles	1—single	3.2%
	2—two	88.6%
	3—three or more	8.2%
Time of crash	1—00:00–06:59	13.3%
	2—07:00–09:29	17.4%
	3—09:30–16:29	44.2%
	4—16:30–18:59	17.1%
	5—19:00–23:59	8.0%
Day of week	0—Weekdays	83.8%
	1—Weekends	16.2%
Road-surface conditions	1—Rain	17.0%
	2—Snow and ice	0.8%
	3—Dry	82.2%
Tunnel length	0—3000 ≥ L > 1000 m	86.5%
	1—L > 3000 m	13.5%
Speed limit	1—<50 km/h	2.9%
	2—50–79 km/h	7.1%
	3—≥80 km/h	90.0%
Number of lanes	1—Two lanes	7.3%
	2—Three lanes	8.9%
	3—Four lanes or more	83.8%
Crash location	1—Zone 1	34.9%
	2—Zone 2	17.9%
	3—Zone 3	47.3%
Least horizontal radius	Continuous variable	
Maximum longitudinal gradient	Continuous variable	

**Table 3:** Results of the Multicollinearity Test.

Model	Standardized Coefficients	T-Stat	Collinearity Diagnostics	
			Tolerance	VIF
Constant		8.834		
Driver’s gender *	0.258	2.803	0.993	1.007
Driver’s age *	0.079	1.894	0.992	1.008
Fatigue *	0.044	2.054	0.957	1.045
Alcohol *	−0.303	−4.491	0.969	1.032
Safety belt used properly *	0.149	2.807	0.988	1.012
Familiarity with vehicle	0.072	2.685	0.976	1.024
Truck body type *	0.075	2.895	0.968	1.033
Overload *	−0.073	−2.716	0.980	1.020
Number of vehicles *	−0.079	−2.020	0.973	1.028
Time of crash *	−0.024	−2.008	0.966	1.036
Day of week *	−0.128	−3.661	0.985	1.016
Road−surface conditions *	−0.052	−2.683	0.757	1.320
Tunnel length *	0.211	5.358	0.908	1.102
Speed limit *	0.127	3.652	0.801	1.248
Number of lanes *	−0.098	−3.776	0.744	1.344
Crash location *	0.044	2.705	0.760	1.316
Maximum longitudinal gradient	0.051	3.075	0.720	1.389
Least horizontal radius	0.047	1.717	0.981	1.018

Note: * Significant at 5% level. VIF: Variance Inflation Factor.

**Table 4:** Test of Parallel Lines.

Model	−2 Log Likelihood	Chi-Square	df	Sig
Null hypothesis	2195.908			
General	2154.036	41.873	66	0.991

**Table 5:** Results of Estimation.

		Estimates	Wald	Adjusted Odds Ratios (95% CI)
Driver factors	Driver’s gender (base: male)			
	Female *	−1.091	7.941	0.336 (0.157,0.717)
	Driver’s age (base: ≥65)			
	≤25 *	−1.142	10.144	0.319 (0.158,0.645)
	26–54 *	−1.224	15.981	0.294 (0.161,0.536)
	Fatigue (base: No)			
	Unknown *	−1.298	33.931	0.273 (0.176,0.423)
	Yes *	0.223	3.994	1.250 (1.004,1.554)
	Alcohol (base: No)			
	Yes *	0.876	10.129	2.401 (1.399,4.116)
	Safety belt used properly (base: No)			
	Yes *	−0.421	3.921	0.656 (0.433,0.996)
Environmental factors	Time of the crash (base: 19:00–23:59)			
	00:00–06:59 *	0.816	12.708	2.261 (1.443,3.543)
	07:00–09:29	0.183	0.745	1.201 (0.792,1.822)
	09:30–16:29	0.203	1.142	1.225 (0.845,1.779)
	16:30–18:59 *	0.442	4.239	1.556 (1.021,2.370)
	Day of week (base: weekends)			
	Weekdays *	0.323	5.667	1.381 (1.059,1.802)
	Road surface Condition (base: Dry)			
	Rain	0.373	5.976	1.452 (1.077,1.960)
	Snow and ice	1.503	5.616	4.495 (1.297,15.580)
Vehicle factors	Truck body type (base: Combination truck)			
	Single-unit truck	−0.208	4.154	0.812 (0.665,0.992)
	Overload (Base: No)			
	Yes	0.217	4.184	1.242 (1.009,1.528)
	Number of vehicles involved (base: three or more)			
	One	1.084	10.021	2.956 (1.511,5.789)
	Two *	0.499	7.225	1.647 (1.145,2.368)
Tunnel factors	Number of lanes (base: four or more)			
	Two *	0.705	9.196	2.024 (1.283,3.190)
	Three *	0.527	7.432	1.694 (1.160,2.472)
	Speed limit (base: ≥80 km/h)			
	<50 *	−0.95	8.275	0.387 (0.203,0.739)
	50–79 *	−0.745	13.216	0.475 (0.318,0.710)
	Crash location (base: zone 3)			
	Zone 1 *	−0.258	4.067	0.773 (0.602,0.993)
	Zone 2 *	−0.653	21.149	0.520 (0.394,0.687)
	Tunnel length (base: extra-long tunnel)			
	Long tunnel *	−0.848	25.99	0.428 (0.309,0.593)
	Maximum longitudinal gradient			
	Least horizontal radius			

Notes: * Significant at 5% level.
